# The effects of bacterial volatile emissions on plant abiotic stress tolerance

**DOI:** 10.3389/fpls.2015.00774

**Published:** 2015-09-24

**Authors:** Xiao-Min Liu, Huiming Zhang

**Affiliations:** Shanghai Center for Plant Stress Biology, Shanghai Institutes for Biological Sciences, Chinese Academy of Sciences, Shanghai, China

**Keywords:** plant growth-promoting rhizobacteria, volatile organic compounds, abiotic stress, salinity, drought, sulfur nutrition, iron deficiency

## Abstract

Plant growth-promoting rhizobacteria (PGPR) are beneficial plant symbionts that have been successfully used in agriculture to increase seedling emergence, plant weight, crop yield, and disease resistance. Some PGPR strains release volatile organic compounds (VOCs) that can directly and/or indirectly mediate increases in plant biomass, disease resistance, and abiotic stress tolerance. This mini-review focuses on the enhancement of plant abiotic stress tolerance by bacterial VOCs. The review considers how PGPR VOCs induce tolerance to salinity and drought stress and also how they improve sulfur and iron nutrition in plants. The potential complexities in evaluating the effects of PGPR VOCs are also discussed.

## Introduction

Plants live naturally with many microorganisms, and the nutrient-rich environment of the rhizosphere is especially conducive to interactions between microorganisms and plants. While many soil microorganisms have no observable effects on plants, others enhance or inhibit plant growth. Plant growth-promoting rhizobacteria (PGPR) are beneficial soil microorganisms that can stimulate plant growth or increase tolerance to stresses. Some PGPR have been applied in agriculture, resulting in increased seedling emergence, plant weight, crop yield, and disease resistance (Kloepper et al., 1980, 1991, 1999). PGPR promote plant growth by producing non-volatile substances, such as the hormones auxin and cytokinin, as well as 1-aminocyclopropane-1-carboxylate (ACC) deaminase, which reduces plant ethylene levels, and siderophores, which facilitate root uptake of metal nutrients (Loper and Schroth, 1986; MacDonald et al., 1986; Glick, 1999; Timmusk et al., 1999). In addition, certain PGPR promote plant growth by emitting volatile organic compounds (VOCs). Microbial VOC emission, which was reported in 2003 by Ryu et al., is now recognized as an important aspect of plant-microorganism interactions (Ryu et al., 2003; Wenke et al., 2010; Blom et al., 2011; Bitas et al., 2013; Farag et al., 2013). VOC emission is indeed a common property of a wide variety of soil microorganisms, although the identity and quantity of volatile compounds emitted vary among species (Effmert et al., 2012; Kanchiswamy et al., 2015).

**FIGURE 1:**
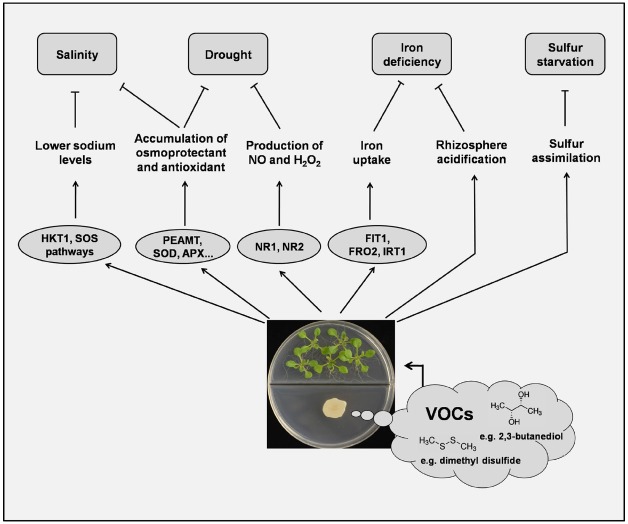
**The effects of microbial volatiles on plant abiotic stress tolerance.** VOCs can modulate *Arabidopsis* sodium homeostasis *via* tissue-specific regulation of *HKT1* and possibly also *via* the SOS pathway. Accumulation of H_2_O_2_ and nitric oxide is involved in the VOC-induced drought tolerance in plants. Accumulation of choline synthesized by VOC-induced *PEAMT* and other osmo-protectants may be a common mechanism for increasing osmotic protection in salt- or drought-stressed plants. VOCs also trigger the expression of *FIT1*, *FRO2*, and *IRT1* to facilitate iron uptake and plant growth. Under conditions of sulfur starvation, plants directly take up and assimilate the S-containing compounds (e.g., dimethyl disulfide) emitted from some PGPR. See text for details.

Although PGPR VOCs do not contain any known plant growth hormones or siderophores (Farag et al., 2013; Kanchiswamy et al., 2015), VOC-mediated regulation of plant endogenous auxin homeostasis and of iron uptake by roots has been documented (Zhang et al., 2007, 2009; Farag et al., 2013). In addition to promoting plant growth, microbial VOCs may also induce disease resistance and abiotic stress tolerance, although the latter phenomenon has been studied only a few times. As a result of the increasing interests in VOCs in mediating plant-microorganism interactions, recent reviews have summarized the chemical nature of microbial VOCs, as well as the effects of microbial VOCs on plant biomass production and disease resistance (Wenke et al., 2010; Bailly and Weisskopf, 2012; Bitas et al., 2013; Farag et al., 2013; Garbeva et al., 2014; Audrain et al., 2015; Kanchiswamy et al., 2015). This mini-review focuses on the enhancement of plant abiotic stress tolerance by bacterial VOCs. In addition to providing an overview of PGPR VOC-induced tolerance to salinity and drought stress, we consider how PGPR VOCs improve sulfur and iron nutrition in plants (Figure 1). Finally, we discuss the potential complexities in evaluating the effects of PGPR VOCs.

## Increased Salt Tolerance

Excessive sodium (Na^+^) creates both ionic and osmotic stresses for plant cells, leading to suppression of plant growth and reduction in crop yields (Zhu, 2001). Bacteria-induced salt tolerance in plants has been observed for several PGPR strains, among which *Bacillus amyloliquefaciens* GB03 (originally described as *Bacillus subtilis* GB03; Choi et al., 2014) displays VOC-mediated effects (Mayak et al., 2004; Barriuso et al., 2008; Zhang et al., 2008a). Salt-stressed *Arabidopsis* plants treated with GB03 VOCs showed greater biomass production and less Na^+^ accumulation compared to salt-stressed plants without VOC treatment (Zhang et al., 2008a). Such VOC-induced stress tolerance was observed in wild-type (WT) plants but not in the *hkt1* null mutant, suggesting a key role of HKT1 in mediating the salt stress tolerance triggered by GB03 VOCs. *Arabidopsis* HKT1 is a xylem parenchyma-expressed Na^+^ transporter that is responsible for Na^+^ exclusion from leaves by removing Na^+^ from the xylem sap (Sunarpi et al., 2005; Horie et al., 2009; Møller et al., 2009). Under salinity stress, GB03 VOCs reduce Na^+^ accumulation in *Arabidopsis* shoots, presumably by enhancing HKT1-dependent shoot-to-root Na^+^ recirculation, because VOCs transcriptionally up-regulate *HKT1* in shoots and concomitantly down-regulate *HKT1* in roots (Zhang et al., 2008a). While it remains unclear how GB03 VOCs regulate *HKT1* transcription, the organ-specific patterns appear to be critical for VOC-induced salt tolerance as well as for auxin-mediated growth promotion (Zhang et al., 2007, 2008a).

GB03 VOCs decreased the Na^+^ level in entire WT *Arabidopsis* plants by approximately 50%, indicating either reduced Na^+^ uptake, enhanced Na^+^ exudation, or both. Intriguingly, GB03 reduced plant Na^+^ levels by only 15% in the *Arabidopsis sos3* mutant (Zhang et al., 2008a). SOS3 is required for post-transcriptional activation of the H^+^/Na^+^ antiporter SOS1, which controls root Na^+^ exudation and long-distance Na^+^ transport in plants (Shi et al., 2000). Therefore, SOS3-dependent Na^+^ exudation is likely required, as a part of the integrated regulation of Na^+^ homeostasis, for the decreased accumulation of Na^+^ in VOC-treated plants. In addition, GB03 VOCs also cause rhizosphere acidification (Zhang et al., 2009), thereby producing a proton gradient that could potentially facilitate the SOS1-mediated export of Na^+^ from roots.

In response to salinity, plants adjust their endogenous metabolism to cope with osmotic stress caused by the excessive accumulation of Na^+^. PGPR-induced salt tolerance was recently reported in soybean plants exposed to volatile emissions from *Pseudomonas simiae* strain AU; the emissions not only decreased root Na^+^ levels but also increased the accumulation of proline, which protect cells from osmotic stress (Vaishnav et al., 2015).Consistent with induced systemic tolerance under salinity, plants treated with AU VOCs showed higher levels of the vegetative storage protein (VSP) and several other proteins that are known to help sustain plant growth under stress conditions (Vaishnav et al., 2015).

## Protection from Water Loss

Dehydration is a common threat to plants experiencing osmotic stress caused by salinity, drought, or cold conditions. Elevated accumulation of osmo-protectants in plants under dehydration stress can increase cellular osmotic pressure to lower the free water potential of cells and thereby prevent water loss, and can also stabilize proteins and membrane structures (Yancey, 1994). Under osmotic stress, *Arabidopsis* exposed to GB03 VOCs accumulated higher levels of choline and glycine betaine than plants without VOC treatment (Zhang et al., 2010). Choline and glycine betaine are important osmo-protectants that confer dehydration tolerance in plants (Rhodes and Hanson, 1993). Consistent with the elevated osmo-protectant levels, plants treated with GB03 VOCs or directly inoculated with GB03 displayed enhanced tolerance to dehydration stress. PEAMT, an essential enzyme in the biosynthesis pathway of choline and glycine betaine (Nuccio et al., 1998; Mou et al., 2002), was suggested to play a key role in mediating VOC-induced plant tolerance to dehydration, because VOC treatment increased the level of *PEAMT* transcripts and because genetic dysfunction of *PEAMT* abolished VOC-induction of dehydration tolerance (Zhang et al., 2010).

GB03 VOCs contain 2,3-butanediol, which promotes plant growth and induces disease resistance (Ryu et al., 2003, 2004). In addition to being found in GB03 VOCs, 2,3-butanediol is also found in the VOCs of some other PGPR strains including *Pseudomonas chlororaphis* strain O6, a bacterium that can trigger induced systemic resistance in plants. Under drought conditions, *Arabidopsis* plants inoculated with *P. chlororaphis* O6 or exposed to 2,3-butanediol exhibited increased stress tolerance, which evidently resulted from increased stomatal closure and reduced water loss (Cho et al., 2008). The application of *P. chlororaphis* O6 or 2,3-butanediol to mutants defective in various hormone signaling pathways indicated that the induced drought tolerance is regulated by multiple classic hormones including salicylic acid (SA), jasmonic acid (JA), and ethylene. In addition, SA appears to play a primary role in the induced drought tolerance, because free SA levels significantly increased in plants treated with *P. chlororaphis* O6 or 2,3-butanediol (Cho et al., 2008). In a subsequent study, 2,3-butanediol was found to induce plant production of nitric oxide (NO) and hydrogen peroxide, while chemical perturbation of NO accumulation impaired 2,3-butanediol-stimulated plant survival under drought stress; these results indicated an important role for NO signaling in the drought tolerance induced by 2,3-butanediol (Cho et al., 2013).

The phytohormone abscisic acid (ABA) is known to control plant stress responses under dehydration conditions. However, the enhanced osmo-protection of plants treated with GB03 VOCs appears to be unrelated to ABA, or at least to ABA production, because osmotic stress caused ABA to increase to similar levels in plants with and without exposure to GB03 VOCs (Zhang et al., 2010). That ABA is not the reason for PGPR-induced plant drought tolerance is further supported by observations that PGPR-treated *Arabidopsis* and cucumber plants accumulated less ABA than control plants (Cho et al., 2008; Kang et al., 2014). An indirect involvement of ABA in such PGPR-triggered abiotic-stress tolerance cannot be completely ruled out, however, given the complex cross-talk among ABA, NO, SA, and hydrogen peroxide signaling pathways in plants (Denancé et al., 2013; León et al., 2014; Song et al., 2014). PGPR-induced drought tolerance can also be mediated through elevated antioxidant responses at the levels of enzyme activity and metabolite accumulation, as was observed in wheat inoculated with *Bacillus safensis* strain W10 and *Ochrobactrum pseudogregnonense* strain IP8 (Chakraborty et al., 2013). Enhanced proline accumulation and gene expression of ROS-scavenging enzymes were observed in PGPR-treated potato plants, which displayed increased tolerance to various abiotic stresses including salinity, drought, and heavy-metal toxicity (Gururani et al., 2013). It would be useful to determine whether volatile emissions enhance antioxidative processes in plants that are stressed by dehydration. Some PGPR strains, such as *Pseudomonas aeruginosa* strain Pa2, produce exopolysaccharides that enhance the ability of the bacteria to maintain soil moisture content and increase drought tolerance in plants (Naseem and Bano, 2014). Certain bacterial VOCs such as acetic acid can induce the formation of biofilms, which contain exopolysaccharides as major constituents (Chen et al., 2015). Thus, it is possible that certain PGPR VOCs may indirectly increase plant drought tolerance by mediating exopolysaccharide production.

## Enhancement of Sulfur Acquisition

As an essential element in many primary metabolites such as the amino acids cysteine and methionine, the macronutrient sulfur (S) is critical for plant survival. Under S-deficient conditions, plants suffer from repression of photosynthesis and disruption of primary metabolism(Burke et al., 1986; Gilbert et al., 1997). While plants mainly acquire S through root uptake of SO_4_^2–^ from soil, plants can also assimilate S from S-containing compounds in the air, including some volatile compounds that are emitted by soil microorganisms (Meldau et al., 2013). Dimethyl disulfide (DMDS) is an S-containing volatile compound commonly produced by many soil bacteria and fungi (Kanchiswamy et al., 2015). Emission of DMDS from *Bacillus* sp. strain B55, a natural symbiont of *Nicotiana attenuata* plants, rescued plant growth retardation caused by S deprivation (Meldau et al., 2013). The incorporation of bacteria-emitted S into plant proteins was demonstrated by adding radio-labeled ^35^S to the bacterial growth medium. In addition to detecting DMDS, Meldau et al. (2013) also detected the S-containing compound S-methyl pentanethioate in *Bacillus* sp. B55 VOCs. The authors attributed most of the S nutrition provided by *Bacillus* sp. B55 VOCs to DMDS rather than to S-methyl pentanethioate for two reasons. First, DMDS was detected as a major component of the volatile emissions while S-methyl pentanethioate was present in only trace amounts. Second, synthetic DMDS was superior to the natural VOC blends in rescuing S-starvation phenotypes of *N. attenuata* plants (Meldau et al., 2013).

Sulfur in SO_4_^2–^ is in an oxidative state and thus requires an energy-consuming reduction process for biological assimilation (Takahashi et al., 2011). In contrast, sulfur in DMDS is in a chemically reduced state. Therefore, it appears that DMDS may not only provide S to plants but may also help plants avoid expending energy on sulfate reduction. Consistent with this hypothesis, DMDS supplementation significantly decreased the expression of S assimilation genes as well as methionine biosynthesis and recycling (Meldau et al., 2013). Like DMDS in *Bacillus* sp. B55 VOCs, other S-containing volatile compounds such as dimethyl sulfide and dimethyl trisulfide have been detected in high concentrations in other microbial VOC blends (Kanchiswamy et al., 2015). Whether these microbial VOCs may also enhance S assimilation by plants remains to be determined.

## Optimization of Iron Homeostasis

The transition between ferrous iron (Fe^2+^) and ferric iron (Fe^3+^) generates a redox potential that is important for electron transfer reactions including photosynthesis. Deprivation of Fe severely impairs the photochemical capacity and is accompanied by leaf chlorosis. Graminaceous monocots produce siderophores that increase Fe^3+^ mobility in soil and directly uptake Fe^3+^ without reduction, while non-graminaceous monocots and dicots not only acidify the rhizosphere to increase Fe^3+^ mobility but also use plasma membrane ferric reductase to reduce Fe^3+^ and subsequently transport Fe^2+^ into roots (Curie and Briat, 2003). Augmented Fe uptake was observed in *Arabidopsis* exposed to GB03 VOCs, which do not contain any known siderophores (Farag et al., 2006; Zhang et al., 2009). Under Fe-sufficient growth conditions, plants treated with GB03 VOCs displayed typical Fe-deficiency responses, including transcriptional up-regulation of the root Fe^3+^ reductase gene *FRO2* and of the Fe^2+^ transporter gene *IRT1*, increases in FRO2 enzyme activity, and rhizosphere acidification (Zhang et al., 2009). As a result, Fe levels were elevated in VOC-treated plants, consistent with greater amounts of Fe-rich photosynthetic apparatus (Zhang et al., 2008b).

GB03 VOC-triggered gene induction of *IRT1* and *FRO2* requires the transcription factor FIT1, because VOC failed to induce *IRT1* or *FRO2* in the *fit1* knockout mutant (Zhang et al., 2009). VOC treatment also failed to increase iron uptake or photosynthesis in the *fit1* mutant. Still, it remains unknown how VOC-treated plants initiate the inducible iron-deficiency responses. One possibility is that a demand for more iron may result from VOC-induced leaf cell expansion (Zhang et al., 2007) and/or photosynthesis augmentation (Zhang et al., 2008b). Also unclear is the identity of the component(s) in GB03 VOCs that induces plant iron-deficiency responses. On the other hand, acid components such as diethyl acetic acid possibly account for the rhizosphere acidification that is directly caused by VOC exposure (Farag et al., 2006; Zhang et al., 2009).

## Potential Complexities of VOC Effects on Plants

Although PGPR VOCs have been shown to benefit plants *via* direct growth promotion, induced resistance to biotic stress, and increased tolerance to abiotic stress (Bailly and Weisskopf, 2012; Bitas et al., 2013; Farag et al., 2013), most of the data concerning these beneficial effects have been obtained in artificial environments. Current studies with PGPR VOCs typically use I-plates (Figure 1), in which a central partition separates plants from bacteria but allows bacterial VOCs to diffuse throughout the plate. This experimental setup appears to favor perception of volatile compounds by leaves, but in natural environments, PGPR VOCs that diffuse through rhizosphere soil pores would mainly be perceived by roots. Therefore, information obtained using I-plates may not apply to natural situations. Another concern with the use of I-plates is that, in addition to releasing volatiles, soil microorganisms also secrete non-volatile compounds (Glick, 1999), which may be taken up by roots and interfere with plant responses to VOCs.

Another complexity in studying the effects of PGPR VOCs on plants is that VOCs from the same PGPR strain may have different effects on plant growth and stress tolerance depending on the nature of the growth medium and the population density of the bacterium (Blom et al., 2011). An increase in the bacterial population in the same space may alter VOC profiles and result in the production of new, toxic components or elevated proportions or quantities of pre-existing toxic components. Alternatively, the VOC component that is responsible for plant growth promotion may accumulate to such high levels that it adversely affects plant growth. Indole, for example, promoted plant growth when applied at low levels but killed plants when applied at high levels (Blom et al., 2011). Similarly, both growth promotion and growth inhibition have been observed for plants treated with DMDS (Kai et al., 2010; Meldau et al., 2013). A change in plant growth conditions may also cause beneficial PGPR VOCs to become inhibitory. GB03 VOCs induce expression of *FIT1* and *IRT1* genes, which enhance plant iron uptake and photosynthesis (Zhang et al., 2009). In addition to transporting iron, IRT1 transports other metal ions such as cadmium into roots (Nishida et al., 2011). It therefore seems possible that GB03 VOCs may aggravate cadmium toxicity in cadmium-stressed plants.

## Conclusion

To date, research on abiotic stress tolerance induced by PGPR VOCs in plants has revealed some interesting phenotypes and initial insights into underlying mechanisms. Nonetheless, further insights into *in planta* molecular mechanisms are needed, especially regarding how VOC signals are perceived by plants and how plants assimilate certain VOC components as metabolites. Future research should also consider the possibility that PGPR VOCs have developed as a consequence of co-evolution. The survival of soil microorganisms is largely dependent on the growth and productivity of the plant community. In addition to supplying leaf litter for decomposers, plants also release up to 30% of their photosynthetic output in the form of root exudates that attract and maintain fungal and bacterial colonies in the soil (Smith et al., 1993; Jones et al., 2003). Therefore, mutually beneficial effects including enhancement of plant abiotic stress tolerance by PGPR VOCs could have resulted from the co-evolution of PGPR with their plant symbionts. Researchers have increasingly recognized that microbial VOCs play important roles in mediating inter- and intra-species interactions. Continued research on PGPR VOCs should lead to improved protection of plants from abiotic stress and to a better understanding of the underlying molecular mechanisms.

### Conflict of Interest Statement

The authors declare that the research was conducted in the absence of any commercial or financial relationships that could be construed as a potential conflict of interest.

## Abbreviations

FIT1: Fe deficiency-induced transcription factor 1
FRO2: ferric reductase oxidase 2
HKT1: high affinity potassium transporter1
IRT1: iron regulated transporter 1
NR1 and 2: nitrate reductase 1 and 2
PEAMT: phosphoethanolamine N-methyltransferase
SOS: salt overly sensitive
VOCs: Volatile Organic Compounds.
